# A Laboratory of Extremophiles: Iceland Coordination Action for Research Activities on Life in Extreme Environments (CAREX) Field Campaign

**DOI:** 10.3390/life3010211

**Published:** 2013-02-25

**Authors:** Viggó Marteinsson, Parag Vaishampayan, Jana Kviderova, Francesca Mapelli, Mauro Medori, Carlo Calfapietra, Angeles Aguilera, Domenica Hamisch, Eyjólfur Reynisson, Sveinn Magnússon, Ramona Marasco, Sara Borin, Abigail Calzada, Virginia Souza-Egipsy, Elena González-Toril, Ricardo Amils, Josef Elster, Robert Hänsch

**Affiliations:** 1 Matis ohf. Food Safety, Environment and Genetics, Vinlandsleid 12, Reykjavik, 113, Iceland; E-Mails: eyjolfur@matis.is (E.R.); Sveinn.h.magnusson@matis.is (S.M.); 2 Biotechnology and Planetary Protection Group, Jet Propulsion Laboratory, California, Institute of Technology, Pasadena, CA 91109, USA; E-Mail: parag.A.Vaishampayan@jpl.nasa.gov; 3 Institute of Botany AS CR, Dukelská 135, Třeboň, CZ-379 82 Czech Republic; E-Mails: kviderova@butbn.cas.cz (J.K.); jelster@butbn.cas.cz (J.E.); 4 Faculty of Science, University of South Bohemia, Branišovská 31, České Budějovice,CZ-370 05, Czech Republic; 5 Department of Food, Environment and Nutritional Sciences (DeFENS), University of Milan, via Celoria 2, Milan, 20133, Italy; E-Mails: francesca.mapelli@unimi.it (F.M.); ramona.marasco@unimi.it (R.M.); sara.borin@unimi.it (S.B.); 6 Consiglio NazionaledelleRicercheIstituto di BiologiaAgroambientale e Forestale via Marconi 2-05010 Porano (TR), Italy; E-Mails: mauro.medori@ibaf.cnr.it (M.M.); carlo.calfapietra@ibaf.cnr.it (C.C.); 7 Centro de Astrobiología. INTA-CSIC. Torrenjón de Ardoz, Madrid, 28850, Spain; E-Mails: aguileraba@cab.inta-csic.es (A.A.); souzaev@inta.es (V.S.-E.); etoril@cbm.uam.es (E.G.-T.); ramils@cbm.uam.es (R.A.); 8 Department of Plant Biology Technical University of Braunschweig, Pockelsstr. 14, Brunschweig, 38092, Germany; E-Mails: d.hamisch@tu-bs.de (D.H.); r.haensch@tu-braunschweig.de (R.H.); 9 Geology Department, University of Oviedo, Jesús Arias de Velasc, Oviedo, 33005, Spain; E-Mail: abigailcalzada@gmail.com

**Keywords:** hot spring, field campaign, interdisciplinary, extreme environment

## Abstract

Existence of life in extreme environments has been known for a long time, and their habitants have been investigated by different scientific disciplines for decades. However, reports of multidisciplinary research are uncommon. In this paper, we report an interdisciplinary three-day field campaign conducted in the framework of the Coordination Action for Research Activities on Life in Extreme Environments (CAREX) FP7EU program, with participation of experts in the fields of life and earth sciences. *In situ* experiments and sampling were performed in a 20 m long hot springs system of different temperature (57 °C to 100 °C) and pH (2 to 4). Abiotic factors were measured to study their influence on the diversity. The CO_2_ and H_2_S concentration varied at different sampling locations in the system, but the SO_2_ remained the same. Four biofilms, mainly composed by four different algae and phototrophic protists, showed differences in photosynthetic activity. Varying temperature of the sampling location affects chlorophyll fluorescence, not only in the microbial mats, but plants (*Juncus*), indicating selective adaptation to the environmental conditions. Quantitative polymerase chain reaction (PCR), DNA microarray and denaturing gradient gel electrophoresis (DGGE)-based analysis in laboratory showed the presence of a diverse microbial population. Even a short duration (30 h) deployment of a micro colonizer in this hot spring system led to colonization of microorganisms based on ribosomal intergenic spacer (RISA) analysis. Polyphasic analysis of this hot spring system was possible due to the involvement of multidisciplinary approaches.

## 1. Introduction

Research on life in extreme environments (LEXEN) has tremendous potential as a source for new bioactive compounds in biotechnology, but it is also essential to understand how life was established on the early Earth and to speculate about the possibilities for life on other planets. Life and growth of living organisms is governed by numerous physical and chemical factors in their environment. Most life forms thrive on the surface of the Earth, where temperatures are generally moderate, *i.e.*, at temperatures from 4 °C to 40 °C, at pH between pH 5 to 8.5 and where salinity, hydrostatic pressure and ionizing radiation are low. Unlike many organisms that cannot survive outside of temperate conditions, extremophiles thrive optimally when one or several of these parameters are in the extreme range [1,2]. Temperature and pH are probably the most drastic factors for growth. Organisms living in such adverse environmental conditions are assigned to thermophilic, psychrophilic, acidophilic and alkalophilic categories. This classification encompasses several natural biotopes in which extreme environmental conditions are more prevalent than usually found in nature. Evidently, considering the high variety of biotopes on Earth, the physiological responses to the environmental extremes can be observed on a gradual scale from tolerance to absolute requirement.

High-temperature environments are generally associated with volcanic activity, but some are also in man-made industrial complexes. Important biotopes are terrestrial geothermal fields, *i.e.*, alkaline freshwater hot springs, acid solfatara fields and hydrothermal systems in marine coastal, shallow and deep areas. Hot environments often display a wide range of pH, from acid to alkaline, depending on temperature, water availability, gases and ion concentrations [3]. Natural geothermal areas are widely distributed around the globe, but they are primarily associated with tectonically active zones at which the movements of the Earth's crust occur. Due to this localization of geothermal heat sources, hot springs are generally restricted to a few concentrated areas. From the biological perspective, the best known terrestrial sites are Iceland, the Naples area in Italy, Yellowstone National Park in USA, Japan, New Zealand and the Kamchatka Peninsula in Siberia [4,5].

Terrestrial geothermal areas, *i.e.*, in Iceland, can be generally divided into high-temperature and low-temperature fields, according to the nature of the heat source and pH. High temperature vent fields are located within the active volcanic zones, and the heat source is a magma chamber at a depth of 2 to 5 km. In these areas, the water temperature reaches 150 °C to 350 °C at the depths of 500 m to 3,000 m, and steam and volcanic gases are emitted at the surface. Mainly, the gases consist of N_2_ and CO_2_, but H_2_S and H_2_ can make up to 10% of the total produced. Traces of CH_4_, NH_3_ and CO can also be found [6]. On the surface, H_2_S is oxidized chemically and biologically first to sulfur and then to sulfuric acid, which acts as the buffering agent in the hot spring environment [1]. As a result, the pH often stabilizes at 2 to 2.5. Because of the high temperature, little liquid water comes to the surface, and the hot springs are usually in the form of fumaroles and steam holes or grey and brown mud pots, resulting from the corrosion of surrounding rocks by the high concentrations of sulfuric acid [6]. Neutral to slightly alkaline sulfide-rich hot springs may also co-exist in high-temperature fields, but are rarer. They appear on the periphery of the active zone and are created if water is abundant at low depths, *i.e.*, by melting of snow or rain or with high levels of the groundwater. The Hveragerdi area in Iceland is a good example of such a field, with a great verity of hot springs with sulfide concentrations as high as 30 mg L^−1^, and under such conditions, thick microbial mats are formed with precipitated sulfur and make spectacular bright yellow or white colors [6,7].

The low temperature hot spring fields are located outside the active volcanic zones. Extinct or deep lava flows and dead magma chambers serve as heat sources, and the water temperature is usually below 150 °C at depths of 500 m to 3,000 m. Groundwater percolating through these zones warms up and returns to the surface, enriched with high concentrations of dissolved minerals (*i.e.*, silica) and gases (mainly CO_2_ and little H_2_S). On the surface, CO_2_ is blown away, and the silica precipitates, resulting in an increase in pH, often stabilizing at 9 to 10. The hot springs in the low temperature field are characterized by a general stability in temperature, water flow and pH [6].

The CAREX project (Coordination Action for Research Activities on Life in Extreme Environments, EC Grant agreement no.: 211700) started in 2008 and was funded by the European Commission in 2009 [8]. The aim of this program was to improve coordination of research on life in extreme environments (LEXEN) and identify the need for the better coordination of LEXEN research. CAREX objectives were focused on establishing interaction, coordinating activities and promoting a community identity for European research in LEXEN. To reach these very ambitious objectives, there is no better way than a real scientific campaign with scientists from different fields of expertise collaborating in a fieldtrip. With this idea in mind, CAREX designed a task, which was called “Field Procedures Inter-comparisons”. One of the main CAREX objectives was to coordinate research interdisciplinary integrated actions as campaigns for studying extreme field sites with multidisciplinary international teams of scientists. Establishing such a community will encourage greater interdisciplinarity and increase knowledge of extreme environments from very different perspectives. This activity was planned to develop fieldtrips; the first of them was organized for a scientific campaign in Río Tinto (South-west Spain), and the second one in Iceland, which is reported in this paper.

The Icelandic field visits were organized in order to promote the interaction of different disciplines in a field setting to demonstrate the use of selected technologies, compare methodologies, exchange research experience and to promote harmonization of techniques and methodologies. The fieldwork aims were focused on developing and evaluating new technologies of common use across LEXEN research, including remote sensing devices and field analysis of ecosystem level processes, focusing on hot spring and glacial techniques.

## 2. Results and Discussion

### 2.1. Site Description, “CAREX Hot Spring”

**Figure 1:**
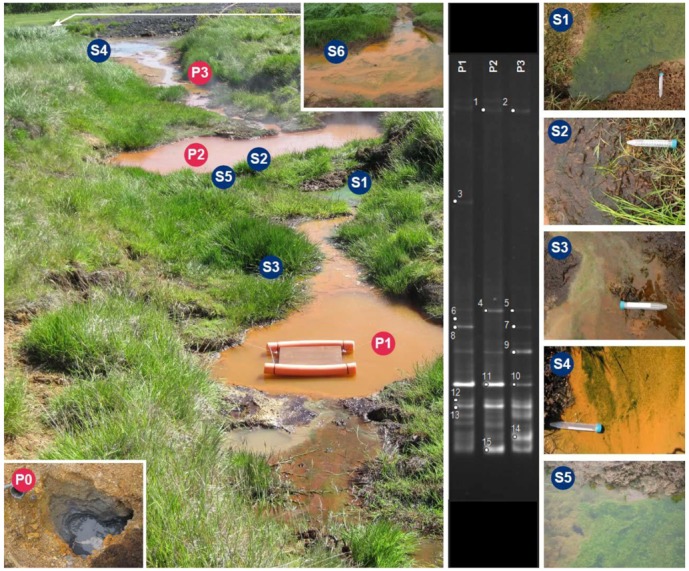
Coordination Action for Research Activities on Life in Extreme Environments (CAREX) hot spring. Picture of the CAREX hot spring system (left photo) and sampling sites, pool 1, 2 and 3, and its specific sampling sites. Different pools of the hot-spring system were sampled: pool 1 (P1), pool 2 (P2), pool 3 (P3) and Pool 0 (P0) (inserted photo in lower left corner). The “Biofilm Catcher” is shown in pool 1 (P1). 16S rRNA polymerase chain reaction (PCR)-denaturing gradient gel electrophoresis (DGGE) profiles of the bacterial communities in the water of the three pools (P1, P2 and P3) are shown on the gel photo in the middle**. **Circles on the bands indicate the DNA fragments that were excised from the gel and successfully amplified and sequenced. Sampling sites for measurements of photochemical activity of microbial mats and higher plants with FluorCam are marked S1, S2, S3 and S4 in the photo of system and enlarged in four photos on the right side of the figure. An enlarged photo of site S5 is also on the right side of the figure (bottom). Site S6 can't be visualized in the photo of the CAREX hot spring and is therefore shown enlarged on the right corner, at the top of the photo.

The sampling site was a high-temperature hot spring field, but with some characteristics of low temperature hot spring fields in Iceland. The sampling zone was comprised of acidic, neutral and alkali hot springs in a narrow area. The formation of these various springs in such a small range was due to abundant water supply in low depths in the surroundings. The selected hot spring in this study was designated as “CAREX hot spring” (Figure 1), which was part of larger system. The hot spring system was about 20 m long with many small spring outlets, which were not visible on the surface, and with different ranges of pH and temperature. The whole system formed three surface inter-connected main pools (P1, P2 and P3) and one open hot spring (P0) at the beginning of the system. This high temperature (98 °C) mud pool (P0) was without any surrounding vegetation and had no surface connection to the nearby pools, P1 or P2 and P3, and it was extremely acidic (pH 3.8). The P system was surrounded by vegetation, and temperature ranged between (57 °C to 62 °C) with a low pH (2.9 to 3.2). Pool P1 was 57 °C with pH 2.9, pool P2 was 62 °C and pH 2.9 and pool P3 was 59 °C and pH 3.2. Two additional hot springs located in the same field, but at approximate 250–300 m away from the CAREX hot spring system, were also measured and used as reference hot springs, HS1 (98 °C, pH 7) and L1 (98 °C, pH 2.0).

### 2.2. Measurements of Photochemical Activity of Microbial Mats and Higher Plants with FluorCam

#### 2.2.1. Measurement in Microbial Mats

The results of *in situ* measurements of photochemical activities of four different microbial mat communities at different environmental conditions are given in Table 1. The microbial mats were collected along the stream at four cooler sites located on the edge of the stream along the CAREX hot spring (Figure 1 S1, S2, S3 and S4).

The following species composition was detected later in laboratory from samples collected from each site (Figure 1 S1 to S4). (Centre for Phycology, Institute of Botany AS CR, Czech Republic).

S1 d_1_—cf. *Chlamydomonas* sp. d_2_—*Klebsormidium* sp., with presence of diatoms, *Euglena* sp.
S2 d_1_—cf. *Zygnematopsis* sp. and d_2_—*Klebsormidium* sp.
S3 d_1_—*Klebsormidium* sp., with presence of diatoms
S4 d_1_—*Euglena* sp., no other species observed

**Table 1:** The environmental conditions at individual sites where microbial mat communities were sampled.

Site	Color of mat	Temperature [°C]	pH	Irradiance [µmol m^−2^ s^−1^]
S1	Green mat	24.9	3.1	460
S2	Brown mat	22.3	3.1	280
S3	Green biofilm	30.5	3.1	550
S4	Green biofilm	24.8	2.7	1,200

Similar species as detected in S1–S4 were also found in an acidic habitat during the CAREX Río Tinto Fieldtrip in Spain, with the exception of *Cyanidium* sp., which dominated in the Río Tinto samples [9]. This study also shows the presence of cf. *Zygnematopsis* sp., which was not found at Río Tinto. Such a difference could reflect the different chemical composition of the water at the study sites, especially heavy metals content [10]. The photochemical performance of the biofilms was evaluated using FluorCam, and the results are summarized in Table 2. Since green algae are dominant in all samples, with the exception of sampling site S4, the parameters could indicate minor stress in the microbial community. Algae in sample S4 was probably photo-inhibited by high irradiance (Table 2). However, for detailed explanations of *in situ* fluorescence parameter measurements, knowledge of the response of individual biofilm species to environmental conditions is crucial, as proposed in Kvíderová [11]. In general, the microbial mats seemed to be well adapted to the given conditions, with the exception of sampling site S4, where the algae were probably subjected to some stress. The stress was probably caused by high irradiance, but low pH effects cannot be excluded. Further laboratory investigations should be performed in defined combinations of temperature, irradiance and pH.

**Table 2:** The photochemical parameters of individual biofilm samples (mean ± SD, n = 3). F_V_/F_M_: maximum quantum yield; Φ_PSII_: actual quantum yield under irradiance of 150 µmol m^−2^ s^−1^; NPQ: Stern-Volmer non-photochemical quenching; qP: photochemical quenching.

	F_V_/F_M_	Φ_PSII_	NPQ	qP
**S1**	0.54 ± 0.02	0.33 ± 0.02	0.09 ± 0.01	0.62 ± 0.03
**S2**	0.64 ± 0.17	0.33 ± 0.04	0.65 ± 0.30	0.63 ± 0.13
**S3**	0.65 ± 0.03	0.26 ± 0.04	0.70 ± 0.04	0.49 ± 0.08
**S4**	0.44 ± 0.00	0.27 ± 0.02	0.07 ± 0.02	0.64 ± 0.04

#### 2.2.2. Measurement in Juncus Plants

**Figure 2:**
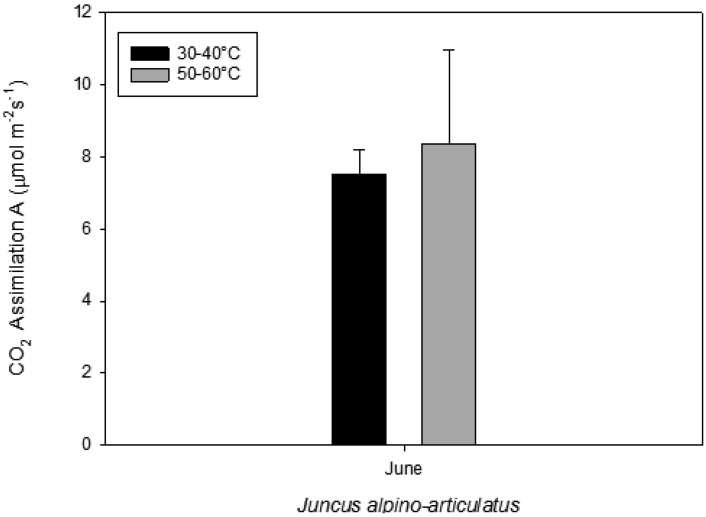
Photosynthesis measured on plants growing near a hot spring at two different temperatures.

Since the F_V_/F_M_ of plants in optimum conditions is approximately 0.83 [12], the F_V_/F_M_ values indicated that the photochemical activity of plants was not seriously damaged by the environmental conditions, and the influence of temperature on photochemical performance was not observed in *Juncus* plants in the CAREX hot spring (Table 3). Other parameters also confirm only minimum stress on the plants. Moreover, FluorCam and Li-COR provided comparable results (Table 3). The photosynthetic apparatus of *Juncus* does not seem to be stressed by high temperatures, and significant differences were found in photochemical parameters derived from fluorescence measurement (F_V_/F_M_, Φ_PSII_, NPQ and qP) and photosynthesis expressed as CO_2_ assimilation rate (Table 3 and Figure 2). However, since the measurements had to be performed in water, the samples drifted during the measurement. The precise evaluation will require step-by-step calculations of individual fluorescence signals from the camera record of the fluorescence. Despite this problem, the results from the automatic data processing by FluorCam software indicate that the plants are not seriously damaged by the environmental conditions, and the influence of temperature on photochemical performance was not observed. 

**Table 3:** Measurements on photochemical performance of *Juncus* plants in the CAREX hot spring (mean SD, n = 3). ). F_V_/F_M_: maximum quantum yield; Φ_PSII_: actual quantum yield under irradiance of 150 µmol m^−2^ s^−1^; NPQ: Stern-Volmer non-photochemical quenching; qP: photochemical quenching.

	30 °C	40 °C	50 °C	60 °C
F_V_/F_M_	0.842 ± 0.017	0.828 ± 0.017	0.845 ± 0.010	0.824 ± 0.021
Φ_PSII_	0.550 ± 0.059	0.584 ± 0.020	0.492 ± 0.132	0.578 ± 0.012
NPQ	0.808 ± 0.229	0.505 ± 0.036	1.288 ± 0.359	0.742 ± 0.311
qP	0.735 ± 0.072	0.768 ± 0.051	0.693 ± 0.166	0.789 ± 0.004

### 2.3. Measurements of Photosynthetic Performance of Thermophilic Biofilms

**Figure 3:**
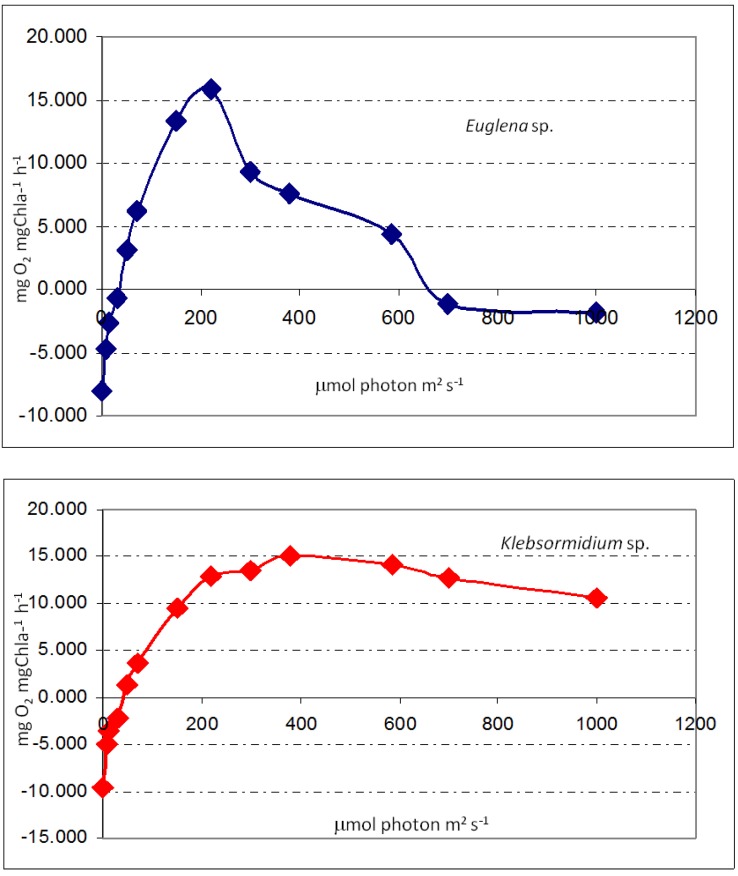
Net oxygen production *versus* irradiance curves. Photosynthetic rates were normalized to chlorophyll *a*. The collection sites for the fluorescence measurements are shown in Figure 1, site S5 and site S6).

Results showed differences in the photosynthetic performance of the biofilms analyzed (Figure 1). While *Euglena* sp. cells (Figure 1, S6 and Figure 3) showed photo-inhibition behavior, the biofilms formed by *Klebsormidium* sp. (Figure 1, S5 and Figure 3) are photo-saturated. *Euglena* sp. showed photo-inhibition over the light intensity of 0 to 200 μmol photons m^−2^ s^−1,^ and the *Klebsormidium* sp. sample showed a light-saturated photosynthesis model under irradiations higher that 200 μmol photons m^−2^ s^−1^. The fitted parameters for the biofilms analyzed are shown in Table 4. All fits showed correlation *r* values higher than 0.85. The highest values of compensation light (Ic) and saturating light (Ik) intensities were shown by *Klebsormidium* sp. biofilm, followed by *Euglena* sp. No significant differences were found in P_max_ values; both biofilms showed *ca*. 15 mgO_2_ mg Chl a^−1^ h^−1^ (Chl a: chlorophyll *a*). However, *Euglena* sp. showed higher photosynthetic efficiency values (α = 0.5) than *Klebsormidium* sp. biofilms (α = 0.24). By sampling two areas (Figure 1, S5 and S6) in the same hot spring system, we obtained different results from two dominating species, and the measurements with FluorCam confirmed our previous results. Furthermore, photosynthetic performance shows photoinhibition in the *Euglena* sp. sample, and no serious damage was detected in *Klebsormidium* sp. in microbial mats samples.

**Table 4:** Photosynthetic parameters of the different biofilms assayed. Compensation light intensity (Ic) and light saturation (Ik) are expressed on a photon basis (µmol photons m^−2^ s^−1^). Maximal photosynthesis rate (P_max_) and photosynthetic efficiency (α) are expressed on a chlorophyll *a* (Chl *a*) basis (mg O_2_ mg Chl a^−1^ h^−1^).

Species	Ic	Ik	Pmax	α
*Euglena* sp.	22.3 ± 2.4	112.6 ± 12.3	16.1 ± 2.6	0.5 ± 0.01
*Klebsormidium* sp.	45.8 ± 4.6	197.6 ± 18.6	15.3 ± 3.1	0.24 ± 0.01

### 2.4. CO_2_ Monitoring at the CAREX Hot Spring

The CO_2_ at the top of the stream at site P0 was measured as 399.6 ppm, P1 was 390.2 ppm and P2 388.7 ppm, on average. The measurement was performed to find out if CO_2_ was in high concentrations and if it was due to volcanic spring activity. CO_2_ is a greenhouse gas naturally present in the atmosphere with a mean concentration of 0.038%. The high value in volcanic areas must either come from geological or biological sources (animals, plants, cells in general). We measured the highest CO_2_ concentration in site P0, which was not vegetated and, therefore, suggests that it was of geological origin.

### 2.5. Volatile Organic Compounds as Carbon Losses in Plants and Their Thermo-Tolerance

**Figure 4:**
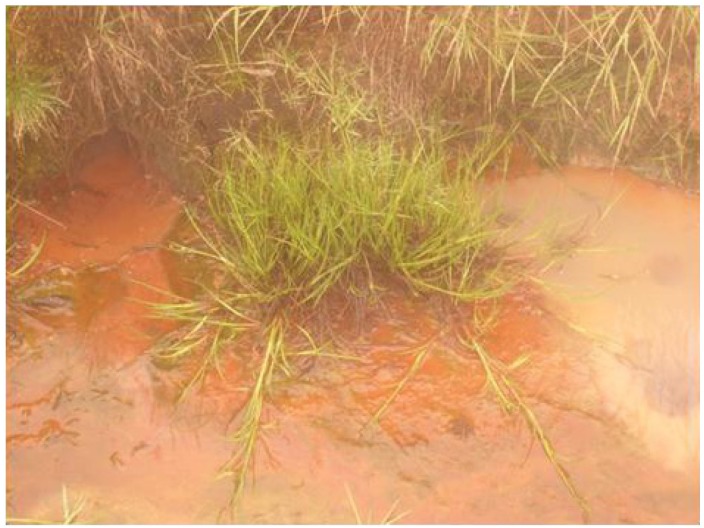
Plant of *Juncusalpino articulatus* growing in water of a temperature between 50 and 60 °C.

**Figure 5:**
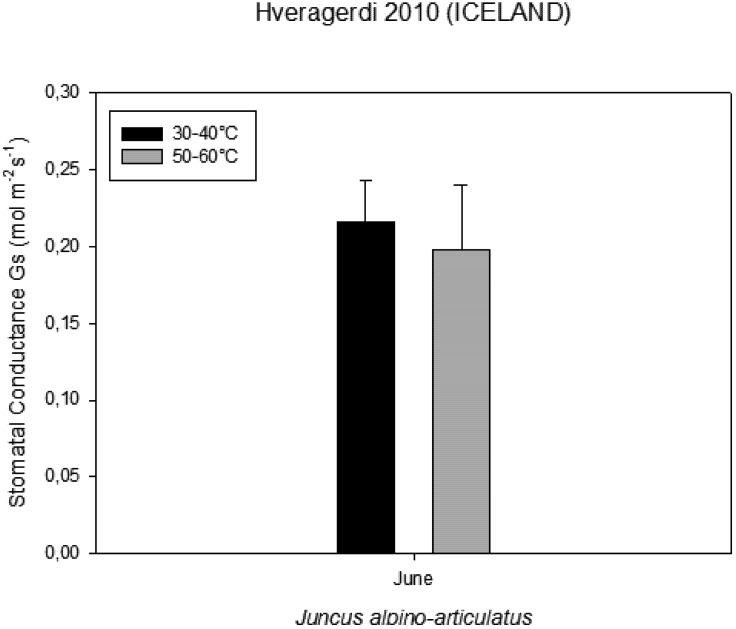
Stomatal conductance measured on plants growing near a hot spring at two different temperatures.

**Figure 6:**
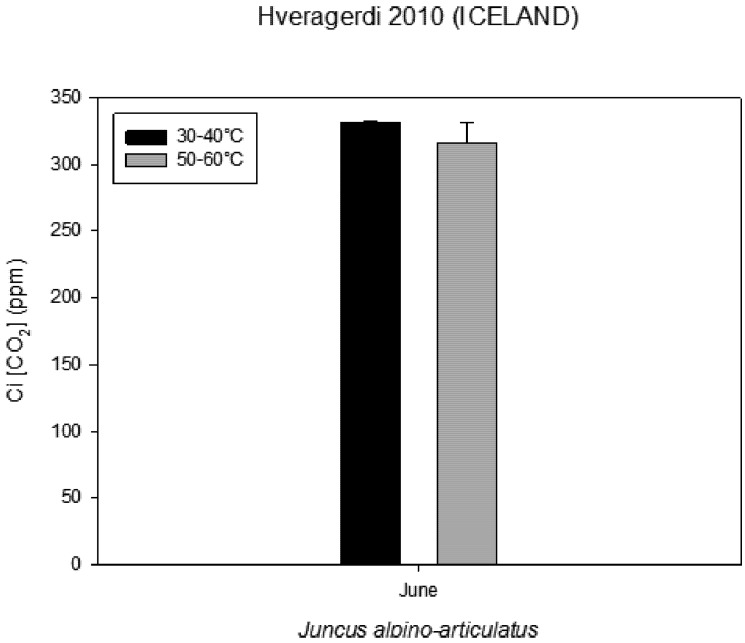
Intercellular CO_2_ concentration (Ci) measured on plants growing near a hot spring at two different temperatures.

**Figure 7:**
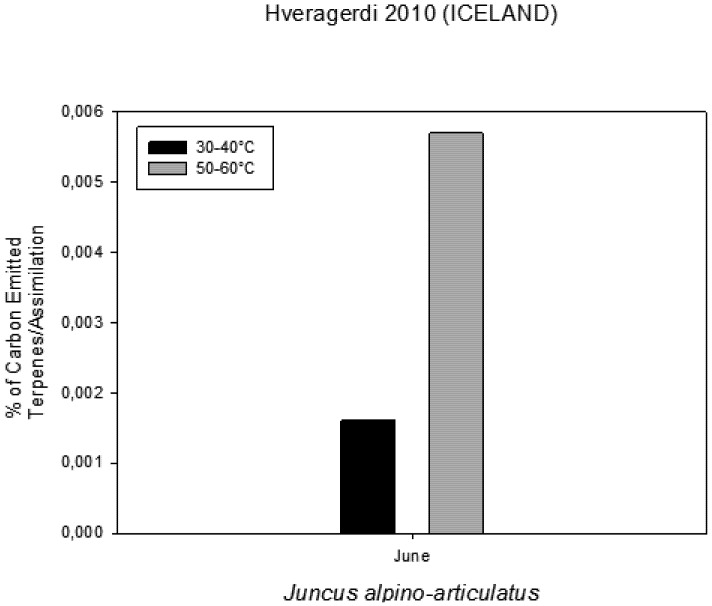
Percentage of Carbon emitted as VOCs in comparison with carbon assimilated through photosynthesis measured on plants growing near a hot spring at two different temperatures.

**Figure 8:**
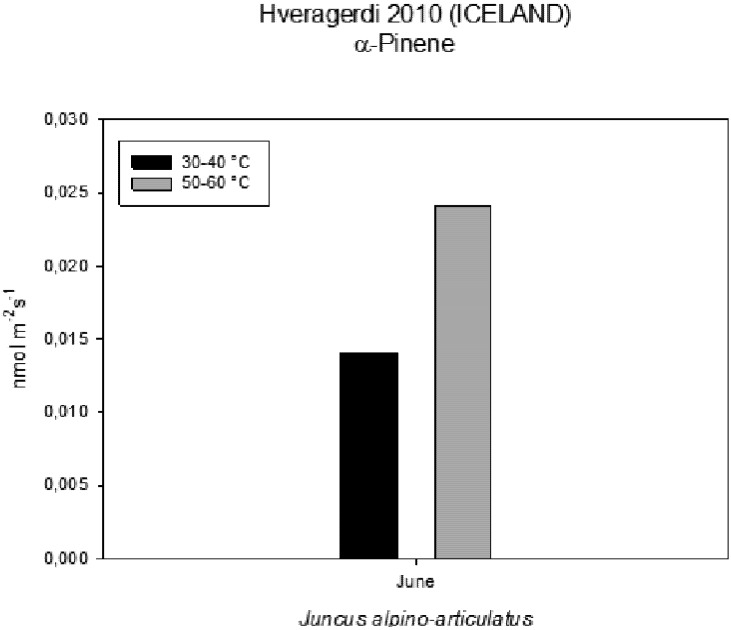
VOCs emitted from plants growing near a hot spring at two different temperatures.

The presence of VOCs in the atmosphere influences its composition and contributes to the formation of greenhouse gases and pollutants [13]. The aim was to investigate the VOC emissions from *Juncusalpino articulatus* living in hot-springs (Figure 4), to identify differences in plants living close to the hot spring from those living further and to relate the VOC emission with the physiological status of the plants. The results are presented in Figure 3, Figure 4, Figure 5, Figure 6, Figure 7, Figure 8 and in more detail by Medori *et al.* [14]. Plants of *Juncus* sp*.* living in higher water temperature (HGT, 50 °C–60 °C) showed a mean value of CO_2_ assimilation of 8.34 µmol m^−^^2^ s^−^^1 ^(Figure 2). This assimilation was higher than that measured in *Juncus* sp. living in lower water temperature (LGT, 30 °C–40 °C) (Figure 4) with the mean value of assimilations was 7.52 µmol m^−^^2^ s^−^^1^ CO2. These high temperatures plants were able to maintain their optimal stomatal conductance (Figure 5). Intercellular CO_2_ concentration (Ci) measured on plants growing near a hot spring at two different temperatures shows stress conditions; in this case, the high water temperatures stimulate plants with greater emissions of VOCs (Figure 6). The rate of carbon emitted with α-pinene represents 0.0057% for HGT and 0.0016% for LGT of the carbon assimilated through the photosynthesis (Figure 7). It is also interesting to find that in both detected species, the emission of VOCs was stimulated by the proximity with the hot spring (Figure 8). Clearly, due to the small number of samples, it is difficult to carry out a statistical test, which could produce reliable results. However it is likely that the warmer temperatures could have stimulated the synthesis of these compounds in plants growing nearby the hot springs, regardless of their protective role in plants. It is known indeed that these compounds are highly dependent on temperature.

### 2.6. SO_2_ and H_2_S Measurements of the CAREX Hot Spring Area

In this study, the control site 50 m away from the study site was measured as SO_2_: 0.0037 ppm and H_2_S: 0,0085 ppm. Site P0 was SO_2_: 0.0111 ppm, H_2_S: 1.8 ppm; site P1 SO_2_: 0.0034 ppm, H_2_S: 0.486 ppm; and site P2 SO_2_: 0.0036 ppm, H_2_S: 0.0138 ppm. The P0 site showed the highest values in both SO_2_ and H_2_S (three- and 35-fold higher, respectively), while the other sites, P2 and P3, had similar values in SO_2_ compared to the control, but they had much higher values in H_2_S, especially P2.

Typical gaseous emissions from geothermal fields and volcanoes are hydrogen sulfide (H_2_S) and sulfur dioxide (SO_2_), which both have influences on human, animal and environmental health. Plants are able to overcome moderate SO_2_ concentrations with sulfite oxidase, a specific enzyme for this purpose: [15]. In volcanic and geothermal areas, most SO_2_ is converted into H_2_S as a result of the prevalently higher pressure: SO_2_ + 3H_2_ ↔ H_2_S + _2_H_2_O [16]. Additionally, it has been reported that SO_2_ generation from H_2_S is minimal and very slow and *vice versa* [17]. Usually, plants gain their sulfur need out of sulfate available from the soil, but further, they are able to use SO_2_ as a sulfur source [18]. Despite the usability of SO_2_, excess amounts are of high toxicity and have influences on the whole plant, up to visible injury and death.

### 2.7. “Biofilm Catcher” in the CAREX Hot Spring, RISA and DGGE Analysis

Water samples collected from the hot spring pools showed higher DNA concentration (ranging from 100 to 200 ng/μL) compared to the different substrates tested by the “Biofilm Catcher”, which displayed low DNA concentration (10–15 ng/μL).

Ribosomal intergenic spacer analysis (RISA) produced faint bands on agarose gel from the pool samples and only from a subset of the substrates samples (paper, iron and titanium) deployed through the “Biofilm Catcher” micro-colonizer, but other solid substrates were negative (pyrite, steel, copper and glass). The RISA profiles showed the presence of a few peaks (Figure 1), indicating the occurrence of a microbiome of low bacterial diversity, both on the pool water and on the “Biofilm Catcher” (data not shown). The RISA profiles showed also the presence of partially different bands among pool water compared to the solid substrates (data not shown), suggesting the selection of specific bacteria on the tested solid materials from the total bacterial community that colonize the P1. However, due to the fact that the retrieved bands were very close to the detection limit of the RISA technique, it is not possible to draw any firm conclusion on the “Biofilm Catcher” experiment. The successful utilization of different solid substrates, including glass, stainless steel and polypropylene, to isolate novel bacteria has been recently demonstrated by inoculating freshwater samples in laboratory microcosms [19]. Possibly, longer periods of deployment of the “Biofilm Catcher” in the natural ecosystem could lead to increased adhesion of the biofilm forming prokaryotes on the solid materials and the selection of previously uncultured bacteria.

Taking in consideration the limits showed by the RISA technique, the bacterial community structure of the pool samples was investigated by DGGE fingerprinting. DGGE profiles obtained from the water samples collected at P1, P2 and P3 (Figure 1) showed very similar bacterial communities in the three interconnected pools, as expected, since the environmental condition are basically the same in terms of pH and temperature (Table 5). Partial 16S rRNA gene sequences (500 bp) obtained from the DGGE bands have been deposited in the GeneBank database under the accession numbers HF547636–HF547650.

**Table 5:** Phylogenetic identification and distribution of bacterial sequences retrieved from 16S rRNA DGGE gel. Identification of the dominant bands in the PCR-DGGE fingerprinting profiles (marked in Figure 1) and their distribution in the three different interconnected pools, P1, P2 and P3, of the hot spring system.

Band	Class (RDP)	Closest Relative (accession number)	%	Environments	Closest Type Strain or Described Cultivable Strain (accession number)	%	Pool		
							P1	P2	P3
12	*Alphaproteobacteria*	Uncultured bacterium (DQ834212)	99	Hot springs, Yellowstone National Park	*Acidicaldus organivorans* (AY140238)	98	**X**	X	X
13	*Alphaproteobacteria*	Uncultured bacterium (DQ834212)	99	Hot springs, Yellowstone National Park	*Acidicaldus organivorans* (AY140238)	99	**X**	X	X
1, 2	*Betaproteobacteria*	*Ralstonia pickettii* (FR873796)	99	Water samples	*Ralstonia pickettii* (AY741342)	99	X	**X**	**X**
15	*Actinobacteria*	*Acidimicrobium* sp. (AY140240)	99	Geothermal sites, Yellowstone National Park	*Acidimicrobium ferrooxidans* (CP001631)	98	X	**X**	X
4, 5	*Bacilli*	*Geobacillus debilis* (AB548612)	99	High temperature compost	*Geobacillus debilis* (AJ564616)	99	X	**X**	**X**
9	*Clostridia*	Uncultured *Bacillus* sp. (EU250948)	89	High temperature compost	*Thermovenabulum ferriorganovorum* (AY033493)	88	X	X	**X**
14	*Clostridia*	Uncultured bacterium (AF523921)	94	Forested wetland	*Sulfobacillus benefaciens* (EF679212)	90	X	X	**X**
6	*Aquificae*	Uncultured *Hydrogenobaculum* sp*.*(EF156602)	99	Norris Geyser, Yellowstone National Park	*Hydrogenobaculum acidophilum* (D16296)	98	**X**		
10, 11	*Aquificae*	Uncultured *Hydrogenophilus* sp. (EF156602)	98	Norris Geyser, Yellowstone National Park	*Hydrogenobaculum acidophilum* (D16296)	97	X	**X**	**X**
3	Unclassified Bacteria	Uncultured *Rhizobiales* (JF317890)	98	Terrestrial hot spring, 85 °C, pH 5.5	*Ignavibacterium album* (AB478415)	85	**X**		
7, 8	Unclassified Bacteria	Uncultured bacterium (EF464600)	96	Acidic mine tailings (pH 3.5-5)	*Thermosinus carboxydivorans* (AAWL01000046)	84	**X**		**X**

%: percent of identity between the DGGE band sequence and the closest relative sequence in GeneBank. Environment: environment of origin of the closest relative sequence. X: presence of the band in the DGGE profile of each sample; in bold are indicated the bands that were actually sequenced.

The identification of the dominant bands in the PCR-DGGE fingerprinting profiles and their distribution in the three different interconnected pools of the hot spring system (P1, P2, P3) are shown in Figure 1. A widely diversified bacterial community composed by different classes of bacteria colonize the three interconnected pools and are represented by *Alphaproteobacteria* (bands 12, 13,), *Betaproteobacteria* (bands 1, 2), *Actinobacteria* (band 15), *Bacilli* (bands 4, 5), *Clostridia* (bands 9, 14) and *Aquificae* (bands 6, 10, 11), besides three bands (3, 7, 8) described as unclassified bacteria. All the obtained sequences showed a high percentage of identity with 16S rRNA bacterial sequences retrieved from environments similar to the CAREX hot spring, such as hot-springs and geysers of Yellowstone National Park.

### 2.8. Adenosine Triphosphate (ATP) Based Analysis

**Figure 9:**
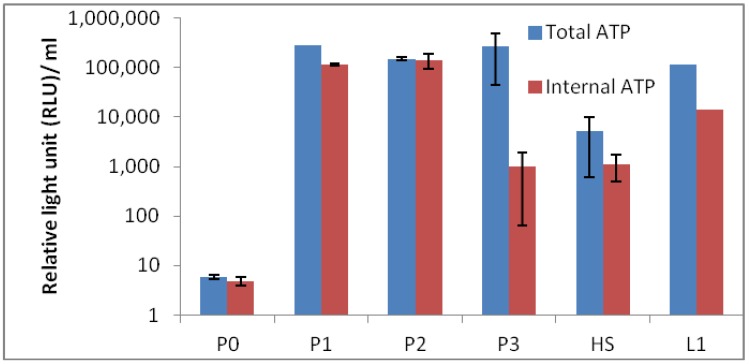
Microbial bio-burden of the hot spring pool samples (P0, P1, P2 and P3) based on total and internal adenosine triphosphate (ATP). HS1 and L1 are samples from reference hot springs.

**Figure 10:**
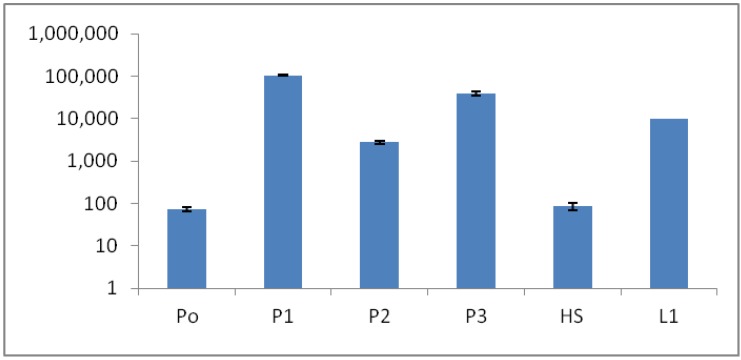
Microbial bio-burden of the hot spring pool samples (P0, P1, P2, P3, HS1 and L1) based on 16S rRNA quantitative polymerase chain reaction (qPCR). Y axis: 16S rRNA copy number/mL.

Results of total and internal adenosine triphosphate (ATP) results are depicted in Figure 9. The results show the expected biomass in each sites and P0 containing the lowest ATP value of them all. The low ATP value at site P0 could be anticipated, as the temperature was high with low pH, and the surroundings were not vegetated. Moreover, the sample at site P0 was expected to be difficult to measure, as clay in the sample was oily and sticky. Therefore, we estimate that the ATP value is in fact underestimated, which was confirmed with measurement based on 16S rRNA quantitative PCR (qPCR) (Figure 10). It is also possible that the clay in the samples was interfering with the qPCR measurements, and therefore, the bio-burden is higher. Lumitester PD-10N is a hand-held ultra-high sensitive ATP measurement instrument, and this lightweight, rapid assay (10 s) instrument has been extensively used by the food industry to monitor microbial bio-burden. The results in this study demonstrate well that this instrument is an ideal field instrument for rapid estimation of bacterial bio-burden to select biological “hot spots” from a large field area.

### 2.9. DNA Extraction and qPCR-Based Bacterial Quantification

The qPCR method measuring the 16S rRNA copy number/mL at the sites (P0 to P3) correlated quite well with the ATP results (Figure 9), although some variance was observed, especially for sites P0 and P2 (Figure 10). These variances could have possibly been explained by some difficulty in DNA extractions, especially from the P0 sample. Bacterial cells can lyse differently, depending on degenerating agents and conditions, but environmental chemicals could also interfere with the DNA yield and PCR performance. The 16S rRNA copy number was significantly lower in P0 and HS1 samples, compared to other sites (Figure 10).

### 2.10. High Density 16S Microarray (PhyloChip) Analysis

The PhyloChip results and other environmental parameters of the sampling sites are shown in Table 6. The Phylochip-based analysis showed the highest diversity of bacteria and archaea at site P1 (313 bacteria and 18 subfamilies of archaea) and P3 (318 bacteria and 18 of subfamilies archaea), but lower diversity at site P2 (127 bacteria and 10 subfamilies of archaea) and very low diversity at site P0 (eight bacteria and zero subfamilies of archaea). More detailed results of the PhyloChip analyses are presented elsewhere by Krebs *et al*. 2013 [20]. The PhyloChip results are in correlation with the ATP and qPCR results, and again, the low diversity at site P2 may be explained by a slightly higher temperature, compared to P1 and P2 samples. The results on archaeal subfamilies detected in the high temperature reference hot springs, HS1 and LS1 (Table 6), show that the PhyloChip technique was sensitive and robust enough for high temperature hot springs by detecting archaea at both neutral pH (7.0) and acidic pH (2.0). Interestingly, the structure of the clay in the acidic reference hot spring, L1, was different from the spring, P0. The clay was brick red in L1, but gray/black in P0, and the clay was more viscous (like oil) in P0 than in L1. The nature of the clay and low water abundance in P0 could possibly explain why low ATP and the 16S rRNA copies were detectable in P0.

**Table 6:** Characteristics of the six pool sites in the CAREX hot spring and two additional reference sampling sites in the same area.

Test	P0	P1	P2	P3	L1	HS1
Temperature (°C)	100	59	64	57	98	98
pH	3.8	3.2	2.9	2.9	2	7
Color of the water	gray/black	brick red	brick red	brick red	brick red	colorless
Presence of Vegetation	no	yes	yes	yes	no	yes
Total ATP	5.8	2.8 x 10^5^	1.5 x 0^5^	2.7 x 10^5^	1.1 x 10^5^	5.3 x 10^3^
Internal ATP	4.8	4.0 x 10^4^	1.4 x 10^5^	1.0 x 10^3^	1.4 x 10^4^	3.7 x 10^2^
Bacterial 16S rRNA copies	7.4 x 10^1^	1.0 x 10^6^	2.8 x 10^4^	3.9 x 10^5^	3.4 x 10^4^	8.6 x 10^2^
*Bacterial subfamilies	8	313	127	318	7	8
*Archaeal subfamilies	0	16	10	18	4	2

* Results based on Phylochips [20].

## 3. Activity Performed on Site and in Laboratory: A Brief Description of Instruments, Materials, Methods and Results.

### 3.1. Sampling Site

*In situ* experiments and sampling were performed in the vegetated hot spring system, designated as CAREX hot spring. The hot spring area of Reykir in Hveragerdi was selected to demonstrate the use of the selected technologies to perform a holistic research on eukaryotic and prokaryotic organisms living in an extreme environment. The research area encompasses various types of hot springs that were formed in May 2008 after a series of strong earthquakes in the area. The zone had low open thermal activity before the earthquakes, which is located a few meters from a small botanical research center in Hveragerdi. This makes the spot very well situated for *in situ* experiments, to interact in a field setting and to demonstrate the use of *in situ* field technologies. The whole thermal active area is located just outside a small town, Hveragerdi, which is located about 50 km east from Reykjavik (Figure 1).

### 3.2. Measurements of Photochemical Activity of Microbial Mats and Higher Plants with FluorCam

The variable chlorophyll *a* fluorescence measurement is a common method in plant physiology (see e.g., [21,22,23] for review of a detailed description of the protocols used). The detailed description of photochemical processes related to individual measured fluorescence parameters is summarized in Gomez *et al.* 2011 [9]. Photochemical activities of microbial mat communities at different environmental conditions and leaves of *Juncus* grass growing in a temperature gradient were investigated. Two biofilm samples from each site were collected. One sample was kept in cold and the second one was fixed using final concentration of 4% formaldehyde + 1% (w/v) CuSO_4_.5H_2_O. The species composition was evaluated using an Olympus BX-51 light microscope (Olympus, Japan) in the laboratory. The photochemical performance of the biofilms was evaluated using a FluorCam fluorescence imaging camera (Photon Systems Instruments, Czech Republic). A quenching protocol was applied for the results, as previously described [9].

### 3.3. Measurement in *Juncus* Plants

The *Juncus* plants were collected in the temperature gradient from 30 to 60 °C. The tips of the leaves were cut, and the fluorescence measurements were performed using the same protocol as for biofilms.

### 3.4. Measurements of Photosynthetic Performance of Thermophilic Biofilms

Light-saturated net photosynthesis (P_max_) was determined as oxygen exchange using an oxygen electrode (Oxytherm, Hansatech Instruments, Norfolk, UK). Two biofilms, mainly composed by filamentous algae, *Klebsormidium* sp., and a phototrophic protist, *Euglena* sp., were analyzed (Figure 1, S5 and S6). Three replicates of each biofilm (*ca*. 1 mg fresh weight) were incubated in the Oxytherm chamber filled with 2 mL of BG11 media at pH 2 at 40 °C and 30 ± 1 °C, respectively, as previously described [24]. Briefly, the biofilms were incubated in darkness for at least 20 min. To estimate P*_max_*, the samples were incubated at different light irradiances ranging from 0 to 1,000 μmol m^−2^ s^−1^ of PAR (µmol m^-2^ s^-1^). Each increase in irradiance was applied, until steady-state oxygen production was observed (5 min). Net photosynthetic rate on the mg Chl *a* basis were determined for each light intensity. Photosynthetic parameters were estimated from the fitting of the equation of Edwards and Walker [25]. The following photosynthetic parameters were estimated by P_max_ (maximum photosynthetic rate under light-saturated condition or photosynthetic capacity), Ic (compensation light intensity), the α value (photosynthetic efficiency) and Ik (light saturation parameter). 

### 3.5. CO_2_ Monitoring at the CAREX Hot Spring

The device used for *CO_2_* measurements was EGM-1 (by PP system, Hitchin, UK), which detects CO2 (ppm) by infrared analysis and is equipped with a chamber for gas sampling from the soil. Each measurement took 2 minutes, and 4 measurements were taken at each point. A reference point was taken in the grassland, about 20 meters south from the stream. The measurements were taken at 1–2 p.m. and the weather was fairly windy.

### 3.6. Contribution of VOCs as Carbon Losses in Plants and Their Thermotolerance

The VOC emissions were measured in 3 plants of *Juncusalpino articulatus* growing at a higher water temperature (between 50 and 60 °C ) and 3 other plants growing at a lower water temperature (between 30 and 40 °C). The sites used for the measurements were located at the end of the CAREX hot spring. Gas exchange was measured using an infrared CO2 and H2O analyzer Li-Cor-6400 photosynthesis system. A leaf area of 6 cm^2^ was measured inside a chamber with a leaf temperature of 20 °C; a photosynthetic photon flux density (PPFD) of 500 μmol m^−^^2^ s^−^^1^, a CO2 concentration of 400 ppm and an air flow through the cuvette of 500 ml/min.

### 3.7. Measurements of the of SO_2_ and H_2_S in the CAREX Hot Spring Area

Measurements of SO_2_ and H_2_S concentrations in the air of the CAREX hot spring were determined by using the APSA 370 air pollution monitor (HORIBA, Kyoto, Japan). Air samples were measured in two zones: the area directly associated to the hot springs (assumed spot of SO_2_ and H_2_S fumigation) and at 20 m distance from the CAREX hot spring, as the control site.

### 3.8. “Biofilm Catcher” in the CAREX Hot Spring, RISA and DGGE Analysis

Water samples were collected from the three inter-connected pools of the CAREX hot spring. About 100 mL of water were filtered from each pool (P1, P2, P3) through 0.22 μm pore size Sterivex filters. DNA extraction was performed directly from the filters using an established protocol [26]. The “Biofilm Catcher” micro-colonizer was deployed in P1 for about 30 hours. Different substrates were used in the micro colonizer in order to obtain the enrichment of biofilm forming bacteria on different solid surfaces (paper, iron, titanium, pyrite, steel, copper and glass). Total DNA was extracted also from the different materials by using a commercial kit (Power Soil DNA Isolation kit) and following the manufacturer's instruction (MoBio, Carlsbad, CA, USA). DNA concentration was evaluated on agarose gel and by NanoDrop spectrophotometer (Thermo Scientific, Wilmington, MA, USA). Ribosomal intergenic spacer analysis (RISA)-PCR was performed as previously described [27] on the DNA extracted from the different tested solid surfaces and on water samples. Denaturing gradient gel electrophoresis (DGGE) was applied on 16S rRNA to describe the hot-spring water dwelling bacterial community by applying the same procedure as reported before [28].

### 3.9. Adenosine Triphosphate (ATP) Based Analysis

Lumitester PD-10N, a hand held ultra-high sensitive ATP measurement instrument (Kikkoman Corporation, Japan), was used to determine the bio-burden of the various sampling locations. The bioluminescence assay was used to determine the total ATP and intracellular ATP of all the samples, as described previously [29,30]. Briefly, to determine total ATP (total microbial population), 0.1 mL sample aliquots (4 replicates) were each combined with 0.1 mL of a cell lysing detergent (benzalkonium chloride) and then incubated at room temperature for 1 min prior to the addition of 0.1 mL of luciferin-luciferase reagent. The sample was mixed, and the resulting bioluminescence was measured with a luminometer. To determine intracellular ATP (total viable microbial population), 0.1 mL of an ATP-eliminating reagent (apyrase, adenosine deaminase) was added to a 1 mL portion of the sample, mixed and allowed to incubate for 30 min to remove any extracellular ATP, after which the assay for ATP was carried out, as described above. As previously established, 1 RLU is approximately equal to 1 colony-forming unit (CFU) [30].

### 3.10. DNA Extraction and qPCR-Based Bacterial Quantification

A matrix of surface water and mud (50 g) was collected in triplicate from each sampling site, and the samples transported from the hot springs site to the laboratory in a cooling box at 4 °C. Nucleic acid from each sample was extracted in duplicate with a Power Soil DNA extraction kit (MoBio), using the manufacturer's protocol. Real-time quantitative polymerase chain reaction (qPCR) assay was performed in triplicate, targeting the 16S rRNA gene to measure bacterial burden with a BioRad CFX-9600 Q-PCR Instrument. Universal bacterial primers targeting the 16S rRNA gene, 1369F (50-CGG TGA ATACGT TCY CGG-30) and modified 1492R (5'-GGW TAC CTTGTT ACG ACT T-3') were used for this analysis [31]. Each 25 μL reaction consisted of 12.5 μL of BioRad 2X iQ SYBR Green Supermix, 1 μL of each of forward and reverse oligonucleotide primer and 1 μL of template DNA. Reaction conditions were as follows: 95 °C denaturation for 3 min, followed by 35 cycles of denaturation at 95 °C for 15 s and a combined annealing and extension at 55 °C for 35 s [20].

### 3.11. High Density 16s Microarray (PhyloChip) Analysis

Bacterial and archaeal 16S rRNA genes were amplified from genomic DNA preparations of each sample, as described earlier. Four separate PCR reactions were performed for each sample with the use of a gradient of annealing temperatures (48 °C, 50.1 °C, 54.4 °C and 57.5 °C). A detailed explanation of the processing of the PhyloChip assay has been described elsewhere [32]. Briefly, the pooled PCR product from each sampling event was spiked with known amounts of synthetic 16S rRNA gene fragments and non-16S rRNA gene fragments. Florescent intensities from these controls were used as standards for normalization among samples. Target fragmentation, biotin labeling, PhyloChip hybridization, scanning and staining, as well as background subtraction, noise calculation and detection and quantification criteria, were performed, as previously reported [33]. An OTU was considered present in the sample when 90% or more of its assigned probe pairs for its corresponding probe set were positive (positive fraction >0.90). For each sample, all OTU intensity measurements were normalized by a scaling factor, such that the overall chip intensity was equal among each PhyloChip [20].

## 4. Conclusions

Very few reports on multidisciplinary field research have been published to date. We report here a successful interdisciplinary research performed in a field campaign with participation of experts in the fields of life and earth sciences. The scientific group successfully selected a hot spring system for ecological studies, such as on environmental factors, chemicals, plants, algae and microbes. The hot spring system was designated as “CAREX hot spring” with a temperature ranging from 30 °C to 98 °C. Measurements of the photochemical activity of microbial mats and higher plants with FluorCam revealed that the microbial mats seemed to be well adapted to the given conditions, but with some exception, as was observed at sampling site S4. Nevertheless, the green algae in S4 were probably stressed by high irradiance, although low pH effects cannot be excluded. The F_V_/F_M_ values indicated that the photochemical activity of the *Juncus* plants was not seriously damaged by the environmental conditions, and the impact of temperature on photochemical performance was not observed. Other parameters obtained with FluorCam and Li-COR also confirm only minimum stress of the plants at these extreme conditions. Moreover, the plants of *Juncus* sp. living in the higher water temperature (HGT, 50–60 °C) showed higher CO_2_ assimilation than that measured in *Juncus* sp. living in the lower water temperature (LGT, 30–40 °C). It is surprising to notice, despite the high temperatures, how plants are able to maintain an optimal physiological state, both in terms of stomatal conductance and assimilation, with basically no decrease of functionality, as compared to the plants grown at lower temperature conditions. It is also interesting to find that in both experimental species, the emission of VOCs was stimulated by the proximity of the hot spring.

The measurements of photosynthetic performance of biofilms were different between species in different sites. While *Euglena* sp. cells showed photo-inhibition behavior, the biofilms formed by *Klebsormidium* sp. were photo-saturated.

The CO_2_ level was significantly higher in the top pool, P0, and less in the other pools. The highest value of CO_2_ was at site P0, which was also not vegetated, and therefore, we suggest that the increased CO_2_ value was of geological origin. The H_2_S concentration was also higher in P0, or 35-fold higher than in other pools, but the SO_2_ remained similar in all of them.

A hand-held instrument was successfully used to measure life without visual observation by detecting *in situ* adenosine triphosphate (ATP) in all samples. This shows the advantage of using such instruments in field campaigns. The ATP results correlated well to the results obtained with quantitative polymerase chain reaction (qPCR). Some microbes attached to the different solid surfaces on the “Biofilm Catcher”, but it was not possible to draw any firm conclusion on the experiment, and a longer incubation time will be necessary for better assumptions. Nevertheless, it is planned to use the “Biofilm Catcher” for long-term exposure experiments in different aquatic ecosystems in the near future.

DGGE profiles obtained from the water samples collected at pools P1, P2 and P3 showed the presence of very similar bacterial communities in the three interconnected pools, and all the sequences showed a high percentage of identity with 16S rRNA bacterial sequences retrieved from similar environments elsewhere. This was anticipated, since the environmental condition between samples was basically the same in terms of pH and temperature. However, deeper analysis of the DNA from the pools is necessary, *i.e.*, with Phylochip, which will be reported independently. The results obtained with the Phylochip shows a much more detailed distinction of the bacterial and archaea taxa, and it reveals the rare microbiota in the samples.

In this study, we have demonstrated that, using polyphasic analysis on a selected environment, the ecology of extremophiles of diverse origin can be studied simultaneously, providing more extensive understanding on the whole ecosystem, rather than focusing on individual life forms separately. Holistic approaches to study ecosystems in a wider perspective are currently lacking in the field, but are important to include in future studies. This research was only a minor effort in that direction, but much more effort is needed.
